# COVID-19 restrictions promoted the newly occurring loneliness in older people – a prospective study in a memory clinic population

**DOI:** 10.3389/fpsyt.2024.1340498

**Published:** 2024-03-11

**Authors:** Michaela Defrancesco, Timo A. Schurr, Alex Hofer

**Affiliations:** Division of Psychiatry I, Department of Psychiatry, Psychotherapy, Psychosomatics and Medical Psychology, Medical University of Innsbruck, Innsbruck, Austria

**Keywords:** dementia, mild cognitive impairment, loneliness, aging, social isolation

## Abstract

**Introduction:**

A high burden and many negative outcomes for older people were associated with the COVID-19 pandemic. Social isolation and loneliness are prevalent health problems impacting well-being and quality of life and may have increased due to pandemic-related restrictions. Methods: This study investigate the influence of the COVID-19 pandemic on loneliness in people visiting a mem40ory clinic between March 2020 and September 2022. We conducted a prospective, single-center, questionnaire-based observational follow-up study to assess potential predictors of newly occurring, pandemic-related loneliness. Next to a newly developed COVID-19 questionnaire, a comprehensive neuropsychological test battery, the Neuropsychiatric Inventory and the Geriatric Depression Scale were used.

**Results:**

In total 426 people (mean age: 76.48 years, 12.9% cognitively intact, 33.1% diagnosed with Mild Cognitive Impairment, 49.8% diagnosed with dementia, and 4.2% diagnosed with depression) completed the COVID-19 questionnaire at baseline and 166 at follow-up. Newly occurring loneliness was indicated by 22.3% of baseline participants and by 24.1% of follow-up participants. Results of logistic regression analysis showed that living alone (OR 5.452) and having less contact with friends (OR 2.771) were most predictive of the occurrence of loneliness. The use of digital communication media as an alternative strategy for social interaction was lowest in dementia patients (6-13%).

**Discussion:**

In conclusion, personal contacts and a close friendship network appear to be more decisive to prevent loneliness in older people than does the use of digital communication media. However, promoting an intensified use of digital communication media may be useful to counteract loneliness, especially in dementia patients.

## 1 Introduction

Starting in 2020, the total number of people affected by the novel coronavirus disease (COVID-19) and the number of associated deaths increased worldwide. Older persons suffering from dementia or cardiovascular diseases were at highest risk of death (1–3). As the pandemic progressed, an increasing number of publications reported worsening cognitive function and neuropsychiatric symptoms in patients with dementia (4–7). Among older adults, public health policy measures such as social distancing may have increased the risk for newly developing mental health disorders (8–11).

Social isolation (an objective measure of missing social relationships) and loneliness (subjective perception of social isolation or “social pain”) are serious but underappreciated public health concerns that are particularly common in older people. Of note, they are associated with numerous negative consequences for this population (12–14). For example, prior studies suggest that social isolation is an important risk factor for Mild Cognitive Impairment (MCI) and dementia (15, 16) and accordingly, the advocacy brief of the World Health Organization (17) concludes that “social isolation and loneliness among older people are growing public health and public policy concerns which have been made more salient by the COVID-19 pandemic”. Pre-pandemic research had already shown that loneliness and social isolation are very common in this population. Being female, living alone, low education and poor mental and physical health have been reported to be important risk factors in this regard (14–16, 18). Data on specific risk factors for newly occurring loneliness during the COVID-19 pandemic, among both cognitively healthy and impaired older people, are scarce.

It is known that loneliness and social isolation are independently associated with poor health outcomes. (19). In line with othes we have, recently shown that the COVID-19 pandemic had a negative impact on the psychological condition of the general population of Tyrol (Austria) and South Tyrol (Italy) and that the degree of loneliness significantly predicted psychological distress in the short-term (20).

Close family relationships and their collaboration with professional caregivers are important aspects of well-being in the lives of older people and people with dementia, and may prevent social isolation and cognitive decline (21–23). Furthermore, numerous studies have reported on the protective effects of social activities as stimuli to increase physical health and cognitive functions in older people (24). Higher levels of social interaction are associated with fewer neuropsychiatric symptoms in this population (25). Accordingly, the negative impact of the pandemic on older adults is largely due to strict COVID-19 action plans, including social and physical distancing, quarantine, and social isolation (26). Limited access to alternative sources of medical and psychological support, such as telemedicine services or digital communication technologies, is another relevant aspect in addition to the lack of face-to-face contact. Older people without dementia, and especially those with dementia, often live alone and use the Internet or social media rather infrequently (27), whereas the use of digital communication media could have a high potential to combat social isolation in late life (28). Although government restrictions have most likely saved lives, the potential negative effects of these restrictions on the well-being of older adults and people with dementia remain unclear. Therefore, prospective and retrospective clinical studies are urgently needed to determine the short- and long-term effects of the COVID-19 pandemic on loneliness and social isolation in older people in general and dementia patients in particular. To fill this gap, the current prospective observational study assessed the prevalence of new-onset loneliness and associated risk factors in outpatients of an Austrian memory clinic during the COVID-19 pandemic. We hypothesized that reduced cognitive, social, and physical activities might have increased this prevalence in older persons in general and especially in those with dementia. Therefore, we investigated whether demographics and numerous social factors associated with COVID-19 restrictions were predictive in this regard. We hypothesized that a detailed evaluation of self-reports as assessed by a questionnaire together with a clinical and neuropsychological examination in our memory clinic would provide comprehensive information about the vulnerable population of older people with cognitive decline. We aimed to gain a deeper understanding of what kinds of changes in personal social networks of older persons and patients with MCI or dementia with newly occurring loneliness experienced. Further, this study aimed to explore the impact of using digital communication media as possible strategy to avoid loneliness.

## 2 Methods

### 2.1 Study design and participants

This was a prospective, single-center, questionnaire-based study. We used a newly developed questionnaire (COVID-19 questionnaire, patient form) to assess the subjective perception of the impact of the COVID-19 pandemic on social life, areas of care, and information seeking.

The study population consisted of elderly persons with a scheduled appointment at the Memory Clinic of the Department of Psychiatry I at the Medical University of Innsbruck for the assessment of memory complaints or as part of their regular routine check-ups. The survey was conducted between 11th May 2020 and 30th September 2022. All study participants received the newly developed COVID-19 questionnaire (for detail see (29)) by mail one week before the scheduled appointment. They were asked to bring the completed questionnaire to the appointment.

Next to comprehensive neuropsychological assessment, rating scales assessing neuropsychiatric symptoms, depression, social and care situation as well as a clinical interview were done as part of standard clinical procedure.

Inclusion criteria comprised an age ≥ 65 years. Individuals were excluded if they were unable to adequately understand the questionnaires due to moderate or severe cognitive impairment, language barrier, or unwillingness to answer the questions. Written informed consent was obtained from the participants and the study was approved by the Ethics Committee of the Medical University of Innsbruck, Austria.

### 2.2 Classification and diagnostic of patient groups

Patients were classified as “Cognitively intact” (CI) if they did not fall below the threshold of 1 standard deviation (SD) below the mean of normative data derived from a representative sample in the neuropsychological test battery and a Clinical Dementia Rating Scale (CDR) (30) score of 0. MCI was diagnosed according to the criteria of Petersen et al. (31), i.e. in patients reporting subjective memory complaints over the previous 6 months and showing impaired memory function (verbal or figural) in the neuropsychological assessment >1.5 SD below the mean of normative data and additionally having a CDR score between 0 and 0.5. Dementia of any etiology (Alzheimer´s dementia (AD), vascular dementia (VD), Dementia due to Chorea Huntington, alcohol-related dementia, Pick’s disease) was diagnosed (ICD-10 criteria) in case of 1) presence of subjective memory complaints over the past 6 months, 2) neuropsychological impairment > 2 SD in one memory function (verbal or figural memory) and at least one other cognitive domain, 3) impairment in activities of daily living as assessed by clinical interview, and 4) a CDR score ≥ 1. For statistical analysis, study participants were assigned to the following diagnostic subcategories: CI, MCI, DEM (including dementia of any etiology).

### 2.3 Power considerations

The power calculation for the primary analysis was conducted with G*Power (version 3.9.2.1) and PASS (version 20) and is based on the type-one error probability of α = 5% and a power of 1-β = 80%. A sample size of 410 participants included will be sufficiently large to detect an OR of 1.62 or higher with a continuous covariate x at the position x ± σ (one standard deviation above/below the mean). This presupposes the assumption, that the p_0_ (probability of loneliness feelings) under the null hypothesis lies in the range of 0.1 to 0.9. Furthermore, it is assumed that the squared multiple correlation among covariates is R² = 0.1. For dichotomous covariates OR ≥ 1.96 are detectable, if the above conditions apply. Hence, the effect sizes to be detected lie in the small to medium range, according to Cohen’s classification (32).

### 2.4 Newly developed COVID-19 questionnaire

The entire survey was related to changes coinciding with the beginning of the COVID-19 pandemic and the start of related restrictions in Austria in March 2020, and the planned visit to the memory clinic. Results of the assessment within the first year of the COVID-19 pandemic have been published previously (29). Briefly, the questionnaire collected general information about the respondent’s living situation (living alone or with a partner or family), marital status, and caregiving situation, as well as the date the questionnaire was completed. Questions on changes in social living (social factors) and on emotional well-being (emotional factors) since the beginning of the COVID-19 pandemic have been rated on a three-part ordinal scale ranging from 0-2 (0 = absent, 1 = sometimes present/occasionally, 2 = frequently present).

### 2.5 Assessment of emotional factors including newly occurring loneliness associated with the COVID-19 pandemic

The questionnaire assessed changes of emotional factors since the beginning of the COVID-19 pandemic and the timing of filling in the questionnaire. It included questions on pandemic-associated changes in emotional symptoms such as loneliness, anxiety, stress and concerns for self and loved ones associated with the COVID-19 pandemic. Study participants were asked to rate every question on emotional factors on the three-part ordinal scale ranging from 0-2. Newly occurring loneliness was assessed by the question: “Did you feel lonely for the first time since the start of the COVID-19 pandemic in March 2020?”. People who reported suffering from occasional or frequent loneliness since the beginning of the COVID-19 pandemic were included in the loneliness “yes” group. Details of the COVID-19 questionnaire for patients on the impact of the COVID-19 pandemic (English translation from German) are presented in S3.

### 2.6 Assessment of social factors associated with the COVID-19 pandemic

The questionnaire assessed changes of social factors since the beginning of the COVID-19 pandemic and the timing of filling in the questionnaire. It included questions about pandemic-related changes, such as face-to-face, telephone, or digital contact with friends and family, participation in events, and the occurrence of disputes. Participants were asked to rate each emotional factor question on a three-point ordinal scale from 0 to 2.

### 2.7 Assessment of neuropsychological functioning

Within the clinical routine at the memory clinic, all study participants completed a comprehensive neuropsychological test battery including subtests of the “Consortium to Establish a Registry for Alzheimer’s Disease” (CERAD) battery (33), as well as the Mini-Mental State Examination (MMSE) (34). Results of the MMSE were used as cognitive measure for statistical analysis.

### 2.8 Assessment of neuropsychiatric functioning and depression

The frequency (range: 0-4 points), severity (1-3 points), and emerging caregiver burden (0-5 points) of twelve neuropsychiatric and behavioral symptoms were assessed using the Neuropsychiatric Inventory (NPI) (35). Depressive symptoms were assessed using the 30-items version of the Geriatric Depression Scale (GDS) (36).

### 2.9 Statistical analysis

Statistical analysis was conducted with R (version 4.2) and IBM SPSS (version 29). The significance level was set to α = 5%. The analytical focus was placed on a comparison of the patients diagnosed as cognitive intact, with MCI, and with dementia regarding demographic, clinical characteristics, time of assessment, loneliness, emotional and social factors. These patient groups were compared by means of Kruskal-Wallis Test for metric variables and Chi-square test for categorical variables. *Post-hoc* comparisons were adjusted by Dunn-Bonferroni method. Additionally, we were interested in investigating differences between patients who reported feeling lonely since the COVID-19 pandemic with those who did not. Therefore, the Mann-Whitney-U test and spearman correlation for metric variables and again, the Chi-square test for categorical variables was employed.

Principal component analysis (PCA) with orthogonal Varimax rotation was used to reduce the number of variables from the social factor questionnaire. The threshold for the Eigenvalue was set to ≥ 1, the threshold for the measure of sampling adequacy was set to ≥ 0.5 for the assessment of individual indicators, the Kaiser-Meyer-Olkin criteria was used to evaluate if the overall data is suitable for PCA. By means of Bartlett’s test, sphericity was tested. Extracted factors were than generated by combining the respective items to a scale, consecutively assessing the scale’s reliability with McDonald’s Omega (ω). Subsequently, the two social scales and item 8 were then entered as independent variables into the logistic regression model.

Initially, a univariate binary logistic regression was conducted, using the dependent variable of newly occurring loneliness “yes” and potential predictor variables (demographic and social factors) as inputs. Independent variables attaining a p-value below 0.15 were also included in the multivariable logistic regression analysis. The goodness-of-fit was assessed with the Hosmer-Lemeshow-test. Reported Odds Ratios (OR) with a 95% confidence interval including a value of 1.0 indicate that there is no association between the respective independent variable and newly occurring loneliness since the start of the COVID-19 pandemic. An OR < 1.0 indicates decreased odds for the occurrence of feeling lonely, whereas an OR > 1.0 indicates increased odds for the newly occurrence of loneliness. For the visualization of the variables effects, a forest-plot was generated.

## 3 Results

Between May 2020 and September 2022, 560 patients meeting inclusion and exclusion criteria came to the Memory Clinic (Department of Psychiatry I) of the Medical University of Innsbruck for an appointment to assess memory complaints or as part of their regular routine check-up. The survey was conducted between 11th May 2020 and 30th September 2022. Out of this sample, 471 individuals (84.1%) completed the COVID-19 questionnaire. Thirty-eight questionnaires were excluded from statistical analysis due to >15% missing items. Finally, the data of 433 individuals (mean age 76.51> 15% years, SD 9.40, range 65-91) could be included in the analyses. Of all people who visited our memory clinic during the study period, a valid response rate of 76.1% was achieved.

Baseline clinical and demographic data of study participants are summarized in **Table 1**. Of the 433 participants 63 (14.5%) were cognitively intact, 158 (36.9%) were diagnosed with MCI, and 212 (48.6%) were diagnosed with dementia (170 AD, 26 VD, 16 dementia with other etiology). Sex distribution, living situation, and marital status were balanced between groups. Age and education were highest and MMSE scores were lowest in the dementia group. The GDS score was highest in the MCI group. The dementia group had the highest NPI scores.

**Table 1:** Clinical and demographic characteristics and social factors of the study sample.

Patients characteristics		Groups			Test statistic^a^	df	*p*-value	*Post-hoc*-test^b^
	Total N=433	CI N=63	MCI N=158	DEM N=212				
	Mean ± SD (%)							
Age (y)	76.51 ± 9.40	71.08 ± 9.98	73.34 ± 10.41	80.49 ± 6.23	*H=*76.838	2	<0.001	DEM*** > MCI, CI
Education (y)	10.79 ± 2.86	11.44 ± 2.44	10.99 ± 3.02	10.45 ± 2.81	*H=*15.402	2	<0.001	DEM*** < CI
MMSE total score	23.76 ± 5.69	29.05 ± 1.36	26.52 ± 2.86	20.05 ± 5.67	*H=*227.011	2	<0.001	DEM*** < MCI***< CI***
GDS total score	9.76 ± 6.27	9.29 ± 6.14	11.25 ± .10	8.78 ± 5.41	*H=*7.582	2	0.023	MCI* > DEM
NPI total score	9.47 ± 9.82	5.36 ± 6.05	8.96 ± 7.72	10.96 ± 11.53	*H=*10.675	2	0.005	DEM**> CI, CI*<MCI
Male	153 (35.3)	25 (39.7)	54 (34.2)	74 (34.9)	χ^2^ = 0.631	2	0.729	
Female	280 (64.7)	38 (60.3)	104 (65.8)	138 (65.1)				
Living situation								
Alone	154 (35.6)	20 (31.7)	58 (36.7)	76 (35.8)	χ^2^ = 0.499	2	0.779	
With partner/family	279 (64.4)	43 (68.3)	9100 (63.3)	136 (64.2)				
Marital status								
Single	41 (9.5)	7 (11.1)	18 (11.4)	16 (7.5)	χ^2^ =10.666	6	0.099	
Married	216 (50.3)	38 (60.3)	81 (51.3)	99 (47.7)				
Divorced/Separated	57 (13.2)	9 (14.3)	21 (13.3)	27 (12.7)				
Widowed	117 (27.0)	9 (14.3)	38 (24.1)	70 (33.0)				
Care situation								
No	228 (25.7)	56 (88.9)	107 (67.7)	65 (30.7)	χ^2^ =99.384	8	<0.001	
Outpatient care	76 (17.6)	2 (3.2)	12 (7.6)	62 (29.2)				
24h care	15 (3.5)	1 (1.6)	2 (1.3)	12 (5.7)				
Day care	4 (0.9)	0 (0)	3 (1.9)	1 (0.5)				
Family care	110 (25.4)	4 (6.3)	34 (21.5)	72 (34.0)				

a Kruskal-Wallis test was used for metric and Chi-square test for nominal variables.

b Dunn-Bonferroni-Test corrected for multiple comparison.

*p<0.05, **p<0.01, ***p < 0.001.

SD, standard deviation; CI, cognitively intact; MCI, Mild Cognitive Impairment; DEM, dementia; DEP, depression; MMSE, Mini Mental State Examination; GDS, Geriatric Depression Scale; NPI, Neuropsychiatric Inventory.

### 3.1 Analysis of non-responders and excluded questionnaires

Of the 560 individuals who were scheduled to visit the Memory Clinic during the study period, 89 (5 CI, 11 MCI, 73 DEM) did not complete the COVID-19 questionnaires but underwent clinical and neuropsychological testing. The most common reasons for not completing the questionnaire were that they had forgotten to fill it out, did not understand the questions, or felt overwhelmed by the questions. A minority of respondents were annoyed by the questionnaire. Responding and non-responding individuals showed no significant differences in terms of cognitive, demographic or social variables. Another 38 questionnaires were excluded from statistical analysis due to >15% of missing items.

### 3.2 New occurrence of loneliness and demographics, cognitive and behavioral symptoms, living and care situation

In total, 98 study participants (22.6%) reported on newly occurring loneliness since the beginning of the COVID-19 pandemic in March 2020.

The group comparison showed significant overall differences for the variables sex, living situation, material status and care situation. Results showed a significantly higher percentage of newly occurring loneliness in women compared to men and in individuals living alone compared to those living with a partner or family. Only 14% of married participants but more than 30% of widowed, single and divorced/separated participants reported newly occurrence of loneliness since the beginning of the COVID-19 pandemic. Study participants with newly occurring loneliness were lower educated and achieved higher scores in the GDS and in the NPI. Care situation, age, and MMSE scores were not associated with newly occurring loneliness. Detailed results are presented in **Table 2**.

**Table 2:** Comparison of demographic, cognitive and behavioral symptoms, living and care situation of study participants with and without newly occurring loneliness.

Patients characteristics	Newly occurring loneliness since the beginning of the pandemic in March 2020		Test statistic^a^	df	p-value
	(yes) N=98	(no) N=335			
	Mean ± SD				
Age (y)	75.29 ± 9.67	76.87 ± 9.31	Z= -1.427	—	0.154
Education (y)	9.82 ± 2.19	11.07 ± 2.96	Z= -3.793	—	<0.001
MMSE total score	23.08 ± 5.89	23.95 ± 5.63	Z= -1.480	—	0.139
GDS total score	12.87 ± 6.74	8.92 ± 5.87	Z= -4.515	—	<0.001
NPI total score	12.09 ± 7.67	8.73 ± 10.23	Z= -4.444	—	<0.001
	N/%				
Diagnosis groups					
CI	9 (9.2)	54 (16.1)	χ^2^ =4.507	2	0.105
MCI	43 (43.9)	115 (34.3)			
DEM	46 (46.9)	166 (49.6)			
Sex					
Male	20 (20.4)	133 (39.7)	χ^2^ =12.352	1	<0.001
Female	78 (79.6)	202 (60.3)			
Living situation					
Alone	63 (64.3)	91 (27.2)	χ^2^=45.592	1	<0.001
With partner/family	35 (35.7)	244 (72.8)			
Marital status					
Single	12 (12.2)	29 (8.7)	χ^2^ =16.177	3	<0.001
Married	32 (32.7)	186 (55.5)			
Divorced/separated	19 (19.4)	38 (11.3)			
Widowed	35 (35.7)	82 (24.5)			
Care situation					
No	42 (42.9)	186(55.5)	χ^2^ =8.824	4	0.066
Outpatient care	23 (23.5)	53 (15.8)			
24h care	6 (6.1)	9 (2.7)			
Day care	0 (0)	4 (1.2)			
Family care	27 (27.6)	83 (24.8)			

a Mann-Whitney U-test was used for metric and Chi-square test for nominal variables.

SD, standard deviation; MMSE, Mini Mental State Examination; GDS, Geriatric Depression Scale; NPI, Neuropsychiatric Inventory.

### 3.3 Newly occurring loneliness and its association with social factors, cognition, depression, and age

A comparison of study participants with vs. without newly occurring loneliness revealed higher scores in all social factors measured in the former group with the exception of “communication via video telephony or social media” and “helping others more often”. Correlation analysis showed that the reporting of a higher frequency of communication via video telephony or social media was associated with higher MMSE scores, higher GDS scores, higher education and lower age. More active phone contacts were associated with a higher MMSE scores and younger age. More disputes with family members or friends was associated with higher NPI scores and lower age. In contrast, helping others more often was associated with lower age, higher MMSE score and higher education. For details see **Table 3** and **Supplementary Table S1**.

**Table 3:** Comparison of newly occurring loneliness and social factors and its correlation with cognition, behavior, mood and demographics.

Question: Did you have the following consequences due to the COVID-19 pandemic starting in March 2020 on an social level?	Newly occurring loneliness since the beginning of the pandemic in March 2020			Mann-Whitney U-test		Spearman correlation with total score				
	total N=433	yes N=98	no N=355			MMSE total score	GDS total score	NPI total score	Age (y)	Education (y)
	Mean ± SD Range 0-2^a^			Test statistic	*p*-value	ρ	ρ	ρ	ρ	ρ
I had less contact with friends	1.27 ± 0.75	1.56 ± 0.64	1.18 ± 0.76	Z =-4.415	<0.001	-0.071	0.042	0.043	-0.091	-0.036
I had less contact with family members	1.01 ± 0.77	1.33 ± 0.78	0.92 ± 0.74	Z =-4.629	<0.001	-0.089	0.035	0.004	-0.066	-0.017
I could not participate in events	0.93 ± 0.86	1.15 ± 0.88	0.86 ± 0.84	Z =-2.905	0.004	0.055	0.064	-0.026	-0.012	-0.088
I helped others more often	0.26 ± 0.54	0.20 ± 0.45	0.27 ± 0.56	Z =-0.748	0.455	0.207***	0.103	-0.009	-0.276***	0.114*
I called others more often	0.74 ± 0.77	0.98 ± 0.82	0.67 ± 0.75	Z =-3.325	<0.001	0.178***	0.080	-0.003	-0.156***	-0.015
I was called less often	0.42 ± 0.64	0.58 ± 0.72	0.38 ± 0.61	Z =-2.38	0.006	-0.033	0.113*	0.015	-0.079	-0.012
I have communicated with video calls and/or social media	0.46 ± 0.74	0.51 ± 0.78	0.44 ± 0.73	Z =-0.774	0.439	0.364***	0.113*	-0.032	-0.487***	0.120*
I had more disputes with family members or friends	0.23 ± 0.52	0.33 ± 0.61	0.21 ± 0.48	Z =-1.968	0.049	-0.056	0.092	0.228***	-0.116*	-0.034
I have felt more social cohesion	0.65 ± 0.74	0.49 ± 0.65	0.69 ± 0.76	Z =2.183	0.029	0.088	-0.033	-0.027	-0.061	-0.030

^a^0 = never, 1 = occasionally, 2 = frequently, *p<0.05, ***p < 0.001.

SD, standard deviation; MMSE, Mini Mental State Examination; GDS, Geriatric Depression Scale; NPI, Neuropsychiatric Inventory.

### 3.4 Group comparison of newly occurring loneliness and other emotional factors

The assessment of loneliness was part of the emotional factor questionnaire. The group comparison showed highest scores of newly occurring loneliness in MCI patients followed by dementia and cognitively intact participants. Group comparisons of participants who rated new-onset loneliness as sometimes or often present vs. never present showed no differences between diagnostic groups. Analysis of the occurrence of different other emotional symptoms showed significant between-group differences in terms of feeling burdened and anxious with highest rates in MCI patients. Further, dementia patients achieved the highest ratings in terms of feeling safe and secure. Details of between-group comparison of newly occurring loneliness and emotional factors are presented in **Supplementary Table S2**.

### 3.5 Correlation of social and emotional factors with time of assessment

The COVID-19 questionnaire was completed 1 to 29 month after the beginning of the COVID-19 pandemic in Austria. 8-25 (mean 15) questionnaires were filled in per month. The regional lockdown policy in Austria included a first hard lockdown from 16 March to 1 April 2020 with gradual easing of restrictions until 1 May 2020, a second "light" lockdown with some restrictions from 21 September 2020 to 3 November 2020, and two further strict lockdowns from 17 November to 6 December 2020 and from 26 December 2020 to 7 February 2021. From 19 May 2021 to 26 July 2022, so-called "3G restrictions" (requirement of either COVID-19 immunization, a negative COVID-19 test, or a recent COVID-19 infection) were active. A strict lockdown was imposed in Austria for a total of 10 weeks. Correlation analysis of social factors with month of assessment revealed a negative correlation of communication via video telephony or social media (Pearsons-correlation, p= 0.021, r = - 0.111) in all diagnosis group and of the feeling of social cohesion only in dementia patients (Pearsons-correlation, p= 0.005, r = - 0.198). Correlation analysis of emotional factors with month of assessment showed a positive correlation of worsening of memory complaints (Pearsons-correlation, p= 0.015, r = 0.193) and the occurrence of nightmares (Pearsons-correlation, p= 0.003, r = 0.232) solely in MCI patients. Reported increase of burden due to the COVID-19 pandemic (Pearsons-correlation, p= 0.046, r = -0.252) showed a negative correlation with month of assessment solely in the cognitively intact participants. All other social and emotional factors as well as loneliness showed no association with time of questionnaire completion.

### 3.6 Principal component analysis of the social factor questionnaire

In total, 401 out of 433 cases were used for the PCA. The measures of sampling adequacy ranged between 0.62 and 0.79, indicating satisfactory values. The Kaiser-Meyer-Olkin criteria was 0.68, Bartlett’s test was statistically significant (χ² (36) = 416.28, p < 0.001). Initially, three components have been extracted (**Table 4**) explaining a total variance of 52.9%. We then calculated the three components scales: scale one consisted of item 1, 2, and 3; scale two consisted of item 4, 5, 6, and 7 and scale three, item 8 and 9. Scale’s reliabilities was not satisfactory for the third scale (ω = 0.170). Next, a two component solution was generated explaining a total variance of 41.5% (**Table 4**). Due to a weak loading on component one, the item 8 has been excluded from the scale building procedure, hence the social factors scale (one) scale one was generated with items 1, 2, 3, and 9; scale two with item 4, 5, 6, and 7, both with satisfactory internal and social factors scale (two) consistency. These two scales and item 8 (after dichotomization) were then used as independent variables within the logistic regression analysis.

**Table 4:** Results of rotated component matrix showing the tree and two component solution of social factors.

Social Factor Questionnaire Items	Component		
	1	2	3
I had less contact with friends (SFS-1) I had less contact with family members (SFS-1) I could not participate in events (SFS-1) I helped others more often (SFS-2) I called others more often (SFS-2) I have communicated with video calls and/or social media (SFS-2) I have felt more social cohesion (SFS-2) I had more disputes with family members or friends I was called less often (SFS-1)	.834 *.845* .782 *.792* .643 *.652* .313 *.339* .265 .483 *.359*	.645 *.644* .623 *.640* .613 *.617* .598 *.586*	*.913* *.485*
Reliability: ω	.681 *.702*	.502 *.502*	*.170*

SFS-1, Social factors scale one; SFS-2, Social factors scale two.

Italic figures represent the three component solution, non-italic figures represent the two factor solution.

### 3.7 Predictors of newly occurring loneliness: results of univariate and multivariable logistic regression analysis

**Figure 1:**
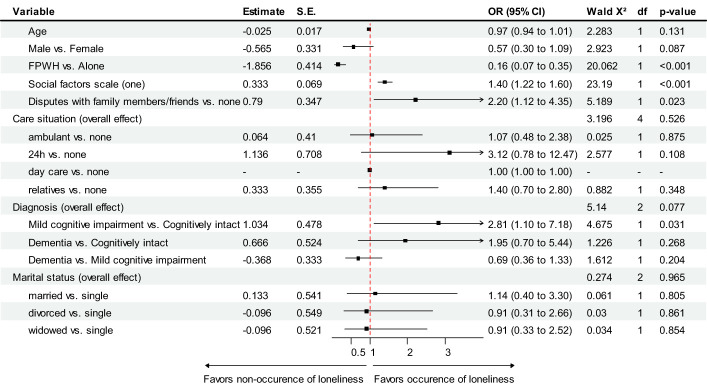
Predictors of newly occurring loneliness following the beginning of the COVID-19 pandemic – results of multivariable logistic regression analysis. FPWH = Living with family, partner or within a residential home, S.E. = Standard error, OR = Odds Ratio, df = degree of freedom, CI = Confidence interval.

Univariate logistic regression analysis showed, that the second social factors scale did not attain a p-value below 0.15 (p = 0.632), therefore it was not included in the multivariable analysis. In total, 410 cases were included in the analysis investigate the joint effect of independent variables on feelings of loneliness. Results including a forest-plot are presented in **Figure 1**. Compared to the null-model there was a statistically significant increment in predictive value due to the inclusion of the independent variables (χ²[15] = 95.68, p < 0.001; Nagelkerke R² = 0.317; correct classification = 81.5%), the goodness-of-fit assessed with the Hosmer-Lemeshow-test (χ²[8] = 9.00, p = 0.343) was satisfactory. Since the “day care” category from the care situation variable included four cases only, the OR obtained in the analysis was undetermined due to almost complete separation [for details see: Heinze, 2006 (37)].

Results indicate that patients living alone are 6.25 times more likely that feelings of loneliness occurred, compared to patients living with their family, friends or within a residential home. Additionally, patients with higher scores on the first social factors scale were more likely to experience loneliness during the measurement period. Having at least sometimes disputes with family members or friends was also associated with higher odds experiencing feelings of loneliness. Although this effect appears to be strong, a wide confidence interval indicates that it could be much weaker or stronger. Marital status, diagnosis and care situation were no significant predictors.

## 4 Discussion

This prospective, observational, questionnaire-based study examined the impact of the COVID-19 pandemic and its limitations on newly occurring loneliness and factors related to social isolation in a memory clinic population between the pandemic beginning in March 2020 and September 2022. To this end, we assessed the perspective of older people with and without cognitive disorders on e.g. social and emotional factors during the first two and a half years of the pandemic. Further, we explored the relationship between the use of digital communication media as an alternative strategy for social interaction and cognitive deficits as well as patients’ living situation.

As expected, clinical and demographic characteristics of the study sample revealed mild to moderate cognitive deficits in approximately 80% of participants according to a diagnosis of MCI or dementia with the highest prevalence of AD. Although the gender distribution was comparable between diagnostic groups, the almost double proportion of women in the MCI and dementia groups is consistent with data from the literature (38). Similarly, a recent report by Livingston et al. corroborates the lowest level of education found in dementia patients (39).

Pre-pandemic studies assessing the prevalence of loneliness in the high aged population are heterogeneous and report that approximately 5% to 50% of individuals aged over 60 experience some degree of loneliness in the course of life (40). In line with these findings, 22.6% of our sample reported newly occurring loneliness since the beginning of the COVID-19 pandemic. Notably, this subgroup was predominantly female, had a lower level of education, lived more often alone, and reported less personal social contacts compared to the remaining group. In more detail, regression analysis revealed that living alone and social factors such as having less contact with the family and friends, less participating in events, and having many disputes with family members or friends since the beginning of the COVID-19 pandemic were significant predictors of newly occurring loneliness. Again, these results are in line with pre-pandemic data that had reported an increased risk for loneliness among older females living alone and having little social relationships (15, 16, 18, 41, 42).

In contrast to our data, most large studies assessing loneliness in the general population in Europe used online surveys and/or did not provide detailed information on clinical data and pre-diagnosed neurocognitive disorders (41–44). A recent meta-analysis of studies conducted in low- and middle-income countries reported an association between MCI and loneliness (45). However, the high rate of loneliness in dementia patients participating in the current study is only partially consistent with the literature (46). Some previous studies found that loneliness is a risk factor for dementia and cognitive decline (47–49), while others did neither find an association between cognitive functioning and loneliness nor higher rates of loneliness e.g. in AD patients (22). Clearly, patients with dementia are particularly prone to loneliness due to cognitive deficits and associated limited possibilities for a number of social activities. Accordingly, the high percentage of newly occurring loneliness in MCI and dementia patients due to the COVID-19 pandemic indicate a reduced ability to develop new coping strategies. It remains to be seen whether interventions which have previously been shown to increase mental health in adults such as supportive text message programs (e.g., Text4Hope) (50) may reduce loneliness and increase well-being in geriatric populations as well.

After the beginning of the pandemic, numerous studies focused on the possible negative impact of public health policy measures on older people (for review see (11, 51)). However, studies assessing the occurrence of loneliness in temporal relation to the beginning of the pandemic are scarce (52–54). Surprisingly, we found no change in the rate of newly occurring loneliness in the course of the assessment period of 29 month. However, MCI patients assessed later in the course of the pandemic reported more frequently nightmares and more memory problems since the beginning of the pandemic. We suggest, that MCI patients are high vulnerable to restrictions such as social distancing. Especially this group of people is still well able to counteract deficits through social, cognitive and physical activities and therefore may have lost a very high number their resources.

Previous studies addressing the fear of dying related to the COVID-19 pandemic are generally rare and have mostly been conducted at the beginning of the pandemic (55) or did not consider the older population (56). Contrary to our expectations, despite the availability of new treatment options and vaccination in the course of the pandemic, the fear of dying was very low in our study population independently of time of assessment and diagnosis. This study found that numerous social factors such as having less contact with family members and friends, less phone calls, and more disputes with family members or friends were associated with the new onset of loneliness in older adults. Social networks have been reported to be important in preventing social isolation and loneliness in people with AD (22) and a recent study found that feeling lonely, especially perceived lack of close relationships, was associated with an 18% increased risk of all-cause mortality in older adults living alone (57). Data of an International Social Survey Program published by Lay-Yee et al. reported that raising social cohesion may prevent loneliness (58), however, it remains to be seen, whether this is also true among patients with dementia.In line with previous publications (59), the use of digital communication media was more frequent in younger and higher educated study participants. We hypothesize that this may be caused by the fact that people with cognitive decline may have hindered the learning of digital alternative strategies for social interaction.

Although digital communication in general has the potential to improve the well-being of older adults, a recent Cochrane meta-analysis reported that the evidence for the effectiveness of digital communication via video calling interventions to reduce loneliness is highly uncertain (60). After the beginning of the pandemic, telemedicine has been shown to be helpful in dementia care. Nevertheless, the elderly population has always been considered “hard to reach” for digital technologies due to lack of interest or cognitive deficits. Our findings suggest that despite technological advances, the use of digital communication media is still not very widespread and is common among older people with cognitive decline. We suggest that digital communication may provide a number of benefits for older people to prevent social isolation.

### 4.1 Limitations and strengths:

One limitation of this study is its retrospective design, in which participants were asked to recall facts or symptoms from the past. Especially in a study population that includes patients with memory deficits, the validity of the data collected in this way may be limited. However, in order to address this recall bias, we excluded study participants with moderate or severe cognitive impairment as well as those with a high percentage of missing items. In addition, the clinical evaluation, cognitive testing, and completion of the COVID-19 questionnaire occurred at approximately the same time, allowing a good estimate of the validity of the data and responses. Another limitation is the single-center design and the inclusion of a highly selected population of memory clinics in one region of Austria. Therefore, our results cannot be generalized to the elderly population in general or to people living in other countries with different restrictions due to the pandemic. Furthermore, the association between new-onset loneliness and both emotional and behavioral symptoms cannot be established causally due to the lack of a control group. The assessment of newly occurring loneliness using a single question and not a validated questionnaire can be seen as a limitation but also as a strength of our study. Validated instruments include the risk of over-complexity for patients with cognitive deficits and we therefore decided to use a limited number of short and easy-to-understand questions directly related to the time of the pandemic to collect data related to different areas of everyday life and emotional and social state.

## 5 Conclusion

The COVID-19 pandemic has a significant negative impact on many areas of everyday life of the older population. Patients with cognitive decline who live alone are at high risk for both loneliness and social isolation, which, in turn, promote the worsening of cognitive deficits and behavioral symptoms. Personal contacts and a close friendship network more than digital communication appeared to be decisive new-onset loneliness in this study. It remains to be seen whether digital communication tools tailored to the individual needs e.g. of dementia patients may be helpful to counteract loneliness and social isolation.

## Data availability statement

The raw data supporting the conclusions of this article will be made available by the authors, without undue reservation.

## Ethics statement

The studies involving humans were approved by Ethics Committee of the Medical University of Innsbruck, Austria. The studies were conducted in accordance with the local legislation and institutional requirements. The participants provided their written informed consent to participate in this study.

## Author contributions

MD: Conceptualization, Data curation, Investigation, Methodology, Project administration, Writing – original draft, Writing – review & editing. TS: Data curation, Formal Analysis, Methodology, Writing – review & editing. AH: Writing – review & editing, Conceptualization, Supervision, Writing – original draft.
