# Supplementary material for: COVID-19 restrictions promoted the newly occurring loneliness in older people – a prospective study in a memory clinic population
Source: Front Psychiatry. 2024 Mar 11;15:1340498. doi: 10.3389/fpsyt.2024.1340498 (PMC10961460; doi:10.3389/fpsyt.2024.1340498)
Supplement: Supplementary file 1 [file Table_1.docx]

Supplement 1. Between-group comparison of newly occurring loneliness and social factors

| Question: Did you have the following consequences due to the COVID-19 pandemic  starting in March 2020 on an social level? | Groups | | | |  |  |  |  |
| --- | --- | --- | --- | --- | --- | --- | --- | --- |
|  | Total  N=433 | CI  N=63 | MCI  N=158 | DEM  N=212 | Test statistic^a^ | df | *p*-value | Post-hoc-test^b^ |
|  | Mean ± SD, Range 0-2^c^ | | | |  |  |  |  |
| I had less contact with friends | 1.27±0.75 | 1.25±0.67 | 1.19±0.74 | 1.33±0.78 | *H=*3.954 | 2 | 0.138 | - |
| I had less contact with family members | 1.01±0.77 | 1.02±0.77 | 0.99±0.74 | 1.02±0.79 | *H=*0.146 | 2 | 0.929 | - |
| I could not participate in events | 0.93±0.86 | 1.11±0.84 | 0.89±0.85 | 0.89±0.86 | *H=*3.381 | 2 | 0.184 | - |
| I helped others more often | 0.26±0.54 | 0.49±0.76 | 0.33±0.56 | 0.13±0.40 | *H=*25.314 | 2 | <0.001 | DEM*** < MCI, DEM*** < CI |
| I called others more often | 0.74±0.77 | 1.03±0.78 | 0.87±0.77 | 0.56±0.73 | *H=*25.563 | 2 | <0.001 | DEM*** < MCI, DEM*** < CI |
| I was called less often | 0.42±0.64 | 0.52±0.71 | 0.43±0.64 | 0.39±0.62 | *H=*1.879 | 2 | 0.391 |  |
| I have communicated with video calls and/or social media | 0.46±0.74 | 0.95±0.85 | 0.77±0.84 | 0.08±0.32 | *H=*117.469 | 2 | <0.001 | DEM*** < MCI, DEM*** < CI |
| I had more disputes with family members or friends | 0.23±0.52 | 0.27±0.55 | 0.19±0.46 | 0.26±0.55 | *H=*1.306 | 2 | 0.520 | - |
| I have felt more social cohesion | 0.65±0.74 | 0.78±0.79 | 0.68±0.71 | 0.59±0.75 | *H=*3.717 | 2 | 0.156 |  |

^a^ Kruskal-Wallis test was used for metric and Chi-square test for nominal variables
^b^ Dunn-Bonferroni-Test corrected for multiple comparison
^c^ 0 = never, 1 = occasionally, 2 = frequently

* p<0.05, ** p<0.01, *** p < .001
Abbreviations: SD =standard deviation, CI= cognitively intact, MCI = Mild Cognitive Impairment, DEM = dementia
