# Supplementary material for: COVID-19 restrictions promoted the newly occurring loneliness in older people – a prospective study in a memory clinic population
Source: Front Psychiatry. 2024 Mar 11;15:1340498. doi: 10.3389/fpsyt.2024.1340498 (PMC10961460; doi:10.3389/fpsyt.2024.1340498)
Supplement: Supplementary file 2 [file Table_2.docx]

Supplement 2. Between-group comparison of newly occurring loneliness and emotional factors

| Question: Did you have the following consequences due to the COVID-19 pandemic  starting in March 2020 on an emotional level? | Groups | | | |  |  |  |  |  |
| --- | --- | --- | --- | --- | --- | --- | --- | --- | --- |
|  | Total  N=433 | CI  N=63 | MCI  N=158 | DEM  N=212 | Test statistic^a^ | df | *p*-value | Post-hoc-test^b^ | |
|  | Mean ± SD, Range 0-2^c^ or (%) | | | |  |  |  |  |  |
| I felt lonely since start of the COVID-19 crisis | 0.61±0.72 | 0.43±0.69 | 0.69±0.75 | 0.64±0.71 | *H=*6.894 | 2 | 0.032 | MCI**>CI, DEM*>CI | |
| Yes^d^ | 98 (22.6) | 9 (9.2) | 43 (43.9) | 46 (46.9) | χ² =4.507 | 2 | 0.105 | - | |
| No | 335 (77.4) | 54 (16.1) | 115 (34.3) | 166 (49.6) |  |  |  | - | |
| I felt burdened due to COVID-19 crisis | 0.84±0.72 | 0.73±0.65 | 0.96±0.73 | 0.78±0.73 | *H=*7.127 | 2 | 0.028 | MCI*>CI, MCI*>DEM | |
| I felt anxious due to COVID-19 crisis | 0.51±0.70 | 0.39±0.64 | 0.63±0.76 | 0.46±0.67 | *H=*6.207 | 2 | 0.045 | CI*<MCI, DEM*<MCI | |
| I mentioned a worsening of memory due to COVID-19 crisis | 0.89±0.80 | 0.68±0.76 | 0.91±0.78 | 0.94±0.83 | *H=*5.086 | 2 | 0.079 | - | |
| I had nightmares due to COVID-19 crisis | 0.21±0.49 | 0.13±0.38 | 0.24±0.52 | 0.20±0.51 | *H=*2.571 | 2 | 0.276 | - | |
| I was afraid of falling ill with COVID-19 | 0.55±0.74 | 0.51±0.62 | 0.60±0.78 | 0.52±0.76 | *H=*1.096 | 2 | 0.578 |  | |
| I had fear of dying from COVID-19 | 0.32±0.65 | 0.21±0.45 | 0.37±0.70 | 0.32±0.66 | *H=*1.549 | 2 | 0.461 | - | |
| I was afraid that my family or friends fall ill with COVID-19 | 0.71±0.76 | 0.65±0.68 | 0.83±0.79 | 0.64±0.75 | *H=*5.789 | 2 | 0.055 | - | |
| I felt safe and secure | 1.17±0.79 | 1.08±0.78 | 1.00±0.79 | 1.28±0.78 | *H=*9.579 | 2 | 0.008 | DEM** > MCI | |

^a^ Kruskal-Wallis test was used for metric and Chi-square test for nominal variables
^b^ Dunn-Bonferroni-Test corrected for multiple comparison
^c^ 0 = never, 1 = occasionally, 2 = frequently
^d^ Yes = item “feeling lonely” rated as occasionally or frequently present

* p<0.05, ** p<0.01, *** p < .001
Abbreviations: SD =standard deviation, CI= cognitively intact, MCI = Mild Cognitive Impairment, DEM = dementia
