# Supplementary material for: COVID-19 restrictions promoted the newly occurring loneliness in older people – a prospective study in a memory clinic population
Source: Front Psychiatry. 2024 Mar 11;15:1340498. doi: 10.3389/fpsyt.2024.1340498 (PMC10961460; doi:10.3389/fpsyt.2024.1340498)
Supplement: Supplementary file 3 [file Table_3.docx]

| Supplement 3: Selection of items of the COVID-19 questionnaire for patients on the impact of the COVID-19 pandemic (English translation from German) | | | | | | | | |
| --- | --- | --- | --- | --- | --- | --- | --- | --- |
| Participant questionnaire | | | | | | | | |
| **Date:** | | | | | | | | |
| **What is your current marital status?** | | | | | | | | |
| Single | | Married | | Divorced/Separated | | | Widowed | |
| **What is your current living situation?** | | | | | | | | |
| Alone | | At home with a partner | | | At home with family | | In a nursing home | |
| **What is your current care situation?** | | | | | | | | |
| no care | outpatient care | | 24h care | | | day care | | family care |
| \| Question: Did you have the following consequences due to the COVID-19 pandemic starting in March 2020 on a social level? \| never/ absent \| sometimes present/ occasionally \| frequently present \| \| --- \| --- \| --- \| --- \| \| I had less contact with friends \|  \|  \|  \| \| I had less contact with family members \|  \|  \|  \| \| I could not participate in events \|  \|  \|  \| \| I helped others more often \|  \|  \|  \| \| I called others more often \|  \|  \|  \| \| I was called less often \|  \|  \|  \| \| I have communicated with video calls and/or social media \|  \|  \|  \| \| I had more disputes with family members or friends \|  \|  \|  \| \| I have felt more social cohesion \|  \|  \|  \| \|  \|  \|  \|  \| \| Question: Did you have the following consequences due to the COVID-19 pandemic starting in March 2020 on an emotional level? \| never/ absent \| sometimes present/ occasionally \| frequently present \| \| I felt lonely since start of the COVID-19 crisis \|  \|  \|  \| \| I felt burdened due to COVID-19 crisis \|  \|  \|  \| \| I felt anxious due to COVID-19 crisis \|  \|  \|  \| \| I mentioned a worsening of memory due to COVID-19 crisis \|  \|  \|  \| \| I had nightmares due to COVID-19 crisis \|  \|  \|  \| \| I was afraid of falling ill with COVID-19 \|  \|  \|  \| \| I had fear of dying from COVID-19 \|  \|  \|  \| \| I was afraid that my family or friends fall ill with COVID-19 \|  \|  \|  \| \| I felt safe and secure \|  \|  \|  \| | | | | | | | | |
